# *In vivo* studies on the biochemical indices of *Plasmodium berghei* infected mice treated with *Alstonia boonei* leaf and root extracts

**DOI:** 10.4314/ahs.v20i4.21

**Published:** 2020-12

**Authors:** Grace C Onyishi, Godwin C Nwosu, Joseph E Eyo

**Affiliations:** Department of Zoology and Environmental Biology, University of Nigeria, Nsukka, Enugu State, Nigeria

**Keywords:** *In vivo*, anti-malaria, *Alstonia boonei*, biochemical, *Plasmodium berghei*, mice

## Abstract

**Background:**

A study on the biochemical indices of albino mice infected with *Plasmodium berghei* and treated with *Alstonia boonei* aqueous and ethanolic extracts was undertaken.

**Methods:**

216 males mice were randomly assigned to six treatment groups each containing six mice for both aqueous and ethanolic extracts experiments. *P. berghei* NK-65 was inoculated into the mice intraperitoneally and establishment of infection confirmed. Administration of extracts of was done after phytochemical and acute toxicity tests at varying concentrations, for both suppressive and curative tests. Blood samples collected by ocular puncturing were examined for the biochemical indices; ALT, AST, ALP, creatinine and total protein using the standard procedures.

**Results:**

*A. boonei* extracts suppression of *P. berghei* in mice was comparable to the standard drug. Significantly higher (p<0.05) recovery of mice treated with A. boonei extracts was observed. The biochemical indices examined all had significantly (p<0.05) increased concentration after 7 days post-infection, except for total protein concentration which had no significant increase or decrease due to *A. boonei* extracts administration.

**Conclusion:**

The antiplasmodial potentials of *A. boonei* leaf and root extracts were dosage and duration-dependent, and have demonstrated satisfactory normalization of altered biochemical indices due to malaria.

## Background

Biochemical indices have been used as a reliable tool for the assessment of the health status of animals^1^. Some biochemical changes that have been reported to occur during malaria include cellular changes in energy metabolism, heme metabolism, membrane lipid peroxidation (LPO) and stress enzymes^2^. Changes in haematological indices (haemoglobin, white cell count, red cell count, mean cellular volume, reticulocyte count, and haemoglobin factor) have been associated with incidences of malaria^3^. Metabolic melee associated with electrolyte and fluid imbalance and changes in the biological functions of the liver are common complications of malaria and are dependent on the level of parasitemia^4^. Alterations in packed cell volume (PCV), random blood glucose (RBG), total bilirubin (TB), total proteins (TP), albumin, serum electrolytes sodium (Na^+^), potassium (K^+^), chloride (Cl^−^), bicarbonate (HCO^3−^), calcium (Ca^2+^), magnesium (Mg^2+^) and anion gap (AG) have been reported in children with malaria^4^. Malaria infections have been established to cause alterations in the plasma biochemical indices^5^. Studies have reported haematological and biochemical changes in malaria parasite-infected blood with common complications associated with this disease. Haematological changes that are associated with malaria infection include anaemia, thrombocytopenia and disseminated intravascular coagulation^6^. Changes in the physicochemical characteristic of *Plasmodium*-infected blood may vary with the degree of malaria endemicity, nutritional status, presence of haemoglobinopathies, demographic factors, and level of immunity to malaria^6^. Thus making haematological parameters essential biomarkers in the assessment of health status.

Furthermore, the infection of cells of the liver by malarial parasite sporozoites can cause cellular inflammation, organ congestion and sinusoidal blockage. These changes in hepatocytes can lead to the leakage of membranous (alkaline phosphatase) and parenchymal (transaminases) liver enzymes to the general body circulation. Hence increase in liver enzymes AST, ALT and ALP observed during malaria episodes also demonstrated that the serum activities of these liver function enzymes increased with the increase *Plasmodium* density. This change could confirm that the hepatic stage of the Plasmodium's life cycle in the animal host is accompanied by significant perturbation in the hepatocyte's parenchyma and membrane leading to leakage of liver enzymes into the general circulation^6^, thus making the assay of liver function enzymes a prerequisite in malaria diagnosis, treatment and management.

Malaria has been reported to be one of the factors responsible for acute renal failure in symptomatic patients in malaria-endemic areas^7^, and the adverse effect of *Plasmodium* on the kidney could lead to increase in blood urea, hypernatraemia, hyperkalaemia, low urine specific gravity, metabolic acidosis and a low ratio of urinary to blood urea^7^. The sudden increase in the urea level and imbalance in the level of the electrolyte such as sodium, potassium, bicarbonate and chloride in malaria-infected persons could serve as indicators for kidney dysfunction^7^ and as such critical factors to be managed during malaria episodes.

The cost of prevention of mosquito bites through the use of mosquito repellants or mosquitocides or treated mosquito nets is high for the inhabitants of malaria-endemic areas all over sub-Saharan Africa. Certain problems hamper malaria management, one of which is the resistance to the most widely available, affordable and safest first-line treatments such as chloroquine and fansider^8^. Secondly, malaria vectors demonstrate resistance to a wide range of insecticides, thereby making vector control strategies difficult. The third and rapidly developing problem is the widespread production of fake anti-malaria drugs and fourthly lack or inadequate infrastructure and resources to manage and control malaria spread and ward off fake curing agents^9^. Clinical trials on a large number of patients showed that artemisinin is effective in clearing parasitaemia and reducing symptoms in patients with malaria, including some with chloroquine-resistant malaria and cerebral malaria^10^. However, artemisinin has a side effect and its use as a monotherapy is not effective. There is a need to search for non-synthetic drugs as a substitute for artemisinin therapy.

*Alstonia boonei* is a large deciduous tree. It is widely distributed in Africa: Cameroon, Central African Republic, Ghana, Code d'Ivoire, Egypt and Nigeria^11^. The chemical, ethnomedicinal, pharmacological and toxicological properties of *A. boonei* has been studied and the result revealed that it is useful in the management and treatment of several illnesses^11^. The root bark is commonly used in Ghana along with other herbs in the management of arthritis^12^,^13^. The anti-inflammatory and antiarthritic properties of the root barks have been reported^14^,^15^. Furthermore, the antioxidant and antimicrobial properties of the stem bark have been documented^16^,^17^. Traditionally in Nigeria, the infusion of the stem bark is drunk as a remedy for snake-bite, and also for arrow poison^17^. It is also used for treating fever and the infusion of the root, stem bark and leaves are drunk as a remedy against asthma^15^. The efficacy of *A. boonei* oil extracts and derivatives as a potential botanical insecticide required for mosquito control has been demonstrated^18^. The blood schizontocidal activity of methanol root bark extracts of *A. boonei* against *P. berghei* infection in Swiss albino mice showed a significant anti-malaria potency which could be exploited in the formulation of standard anti-malaria drug^19^. Studies on ethanolic leaf^5^, methanol root bark^19^, aqueous leaf^23^, methanol stem and leaf^30^, and lime leaf^36^ extracts of *A. boonei* and its effect on some haematological and biochemical parameters of albino mice infected with *P. berghei* have been conducted. None of the studies utilized aqueous and ethanolic leaf and root extract of *A. boonei*. The present work evaluated in vivo the anti-*Plasmodium* and biochemical effects of *A. boonei* root and leaf ethanolic extracts in *P. berghei* infected albino mice. The root and leaf of the plant were selected for extraction because of the reported rich phytochemical composition of this plant parts^11^–^17^.

## Methods

### *Alstonia boonei* extracts and artesunate

The plant leaves and roots were collected from Inyi in Enugu-Ezike (Igbo-Eze North Local Government Area) of Enugu State, Nigeria from the wild. The plant parts were identified and authenticated by a botanist at the Department of Plant Science and Biotechnology, University of Nigeria Nsukka, wherein the voucher specimens (PSBH 2017-141) were kept in their herbarium. Fresh leaves and roots of *A. boonei* were washed, sliced and air-dried separately at room temperature to a constant weight. Each of them was pulverized and 800 g of the leaves and roots fine powder obtained was divided into two. Half of the separate fine powders were percolated in 1400 ml of water and the other half in 70 % ethanol, for aqueous and ethanolic extracts, respectively. They were filtered after 72 hours and the filtrates evaporated to dryness using a temperature-regulated water bath preset at 40° C and the wet granules dried in a hot air oven at 60° for 1 hour. Thereafter, the dried granules were milled and screened through a 1.0 mm sieve to yield the dry powder extracts concentrates. These were labelled accordingly and stored in a refrigerator at 4°C before use. The ethanol and aqueous extracts of *A. boonei* re-suspended in 5 ml of distilled water corresponding to the dosages 200, 400 and 800 mg kg^−1^ b.wt. of mice and the standard antimalarial drug -artesunate (100 mg kg^−1^ b.wt. of mice) were administered by oral intubations. Distilled water (not Tween 80) was used because in Nigeria traditional medicine practitioners use water and alcohol infusion of *A. boonei* to cure various diseases^16^,^17^.

### Acute toxicity (LD_50_) of *Alstonia boonei* extracts

Acute toxicity (LD_50_) of the aqueous and ethanol extracts of A. boonei leaves and roots were determined by Lorke's method^20^. Eighteen (18) adult albino mice were used for this experiment. The experiment was conducted in two phases. In the first phase, three groups of three mice each were orally administered 10, 100, 1000 mg/kg body weight of the extract, respectively and observed for 24 hours for some death and behavioural changes. In the second phase, based on the fact that no mortality was recorded (100 % survival rates), increased doses of 1500, 3000 and 6000 mg/kg body weight were orally administered to three additional mice for each group, respectively, and the fourth mice received only solvent (5% Tween 80) which served as the control. The mice administered with ethanol and aqueous extracts of *A. boonei* leaves and roots were observed for 24 hours and the number of deaths was recorded. The second experiment recorded no death even at 6000 mg/kg, thus the extract was regarded as safe. The least sub-lethal dosage used for this experiment was thirty times reduction of the highest safe dosage and subsequently doubled for increasing dosages.

### Experimental animal

Two hundred and sixteen (216) male conventional grade UN-FERH: NS outbred strain of albino mice (*Mus musculus*) used in the study were procured from the Genetic and Breeding Laboratory of the Department of Zoology and Environmental Biology, University of Nigeria, Nsukka. The mice were maintained according to the National Research Council guidelines on laboratory animal use^21^. Furthermore, this experiment was designed following the Three R (replacement, reduction and refinement) alternatives ethic of animal experimentation^22^, with much emphasis on refinement as the experimental procedures adopted minimized pain, distress, and enhanced the welfare of mice used in this study. Food and water were available *ad libitum*.

*Plasmodium berghei*

The artesunate-sensitive strain of the rodent parasite *Plasmodium berghei* NK-65 was obtained from our institution Veterinary Teaching Hospital. The strain was maintained in the laboratory for the period of the study by in vivo serial blood passage from mouse to mouse^23^. A set of mice parasitized with *P. berghei* NK-65 were anaesthetized after 6 days having shown clinical symptoms of malaria and confirmed microscopically (>2 × 10^7^ *P. berghei* parasitized erythrocytes). Samples of blood were collected by cardiac puncture using a sterile needle and syringe. The samples were diluted in normal saline (1 ml of blood in 10 ml of normal saline), and 0.2 ml of blood containing 1 × 10^7^ *P. berghei* infected erythrocytes was used to infect each of the experimental mice intraperitoneally^19^.

### Study design and anti-plasmodial effects of plant extracts

This study adopted a completely randomized design. Six treatment groups containing six mice each in three replicates for each extract was used in the present study. Group I: Baseline, mice not infected and not treated; Group II: Control, infected and not treated; Group III: Artesunate, infected and treated with the standard drug (artesunate, 100 mg/kg body wt/day); Groups IV, V and VI: 400, 600 and 800 mg/kg body wt/day, infected and treated with 400, 600 and 800 mg/kg/day of extract. Three mice from each group were used for the suppressive test (treated 4 hours after parasite inoculation for 4 days). The remaining mice were used for the curative test (treated 72 hours after parasite inoculation for 4 days) as well as for biochemical examination. At the end of the experiment, their mean survival times (MST) were estimated^24^.

MST = Number of days survived / Total Number of days (28) × 100

### Blood sampling and assay

Mice were anaesthetized by chloroform inhalation in the air-tight chamber. Blood samples (5 ml) collections were made at both tail and ocular regions at three periods (24 hours before parasite inoculation; 72 hours after parasite inoculation; and 24 hours after overall treatment for 4 days). Blood samples were collected from the tail of each mouse to make thin blood smear following standard procedure, and parasitaemia levels were determined microscopically^25^. From the parasitaemia level, percentage parasitaemia and suppression were deduced^25^,^26^.

% Parasitaemia = Total number of parasitized erythrocytes x 100 / Total number of erythrocytes

% Suppression = Parasitaemia in the control group - Parasitaemia in study group × 100 / Parasitaemia in the control group

Also, pooled mouse blood samples collections by ocular puncturing were dispensed into plain bottles for biochemical examinations using the methods of Sood^27^. Samples in plain bottles were centrifuged at 3000 rpm for 10 minutes at room temperature, and biochemical indices of ALT, AST, ALP, creatinine, and total protein were estimated using Randox Diagnostic Kits as described by Reitman and Frankel^28^, except for total protein and creatinine that were estimated using the methods of Bradford^29^. At the end of the experiment, mice were humanely sacrificed under anaesthesia and dead mice incinerated.

## Statistical analysis

Data were analyzed using SPSS version 20. Analysis of Variance (ANOVA) and Duncan's multiple new range test was used to compare the mean differences among extracts concentrations. Mean difference of p<0.05 was regarded as significant.

## Results

### *Alstonia boonei* extracts did not affect the MST of mice

**Figure 1:**
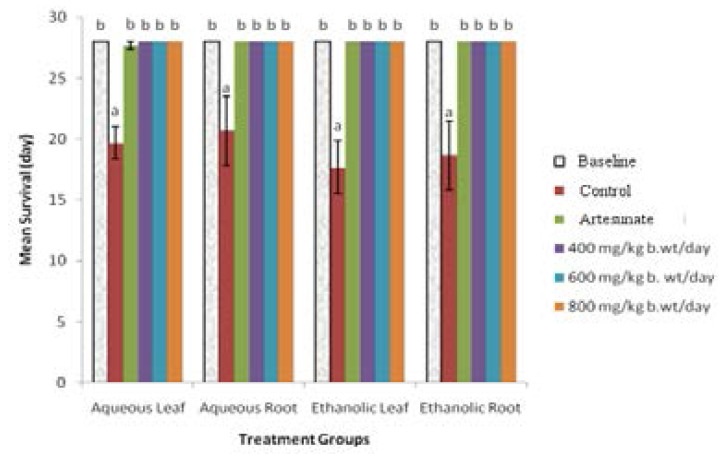
Mean survival time of mice infected with *P. berghei* and treated with leaf and root extracts of *A. boonei* (curative). *Mean values with different alphabets as superscripts are significantly different (p<0.05)*

The comparative survival time of infected mice 28 days post-infection with *P. berghei* and treated with aqueous and ethanolic leaf and root extracts of *A. boonei* had statistically similar (p>0.05) survival time with those treated with the standard drug (Figure 1). Mean survival for the mice infected with *P. berghei* and treated with extracts as well as the standard drug was significantly higher than the MST of infected and untreated mice (p<0.05).

### *Alstonia boonei* extracts suppressed parasitaemia

The percentage parasitaemia and suppression of parasitaemia in *P. berghei* infected mice when treated with aqueous and ethanolic leaf and root extracts of A. boonei are summarized in Tables 1 – 2. The percentage of parasitaemia and parasitaemia suppression for the duration of treatment is presented for both the suppressive and curative tests. Table 1 showed the percentage parasitaemia and average percentage reduction of parasitaemia when treatment was commenced 4 hours post-infection for the suppressive test. The aqueous leaf, ethanolic leaf and ethanolic root extracts at concentration 400, 600 and 800 mg/kg body wt/day had similar parasitaemia suppressive ability comparable to the standard drug (Table 1). This suppressive ability was concentration-dependent, increasing as concentration increases. For the curative test, the percentage parasitaemia and suppression of *A. boonei* extracts on mice infected with *P. berghei* were represented in Table 2. Table 2 showed percentage parasitaemia in mice infected with *P. berghei* at the end of day 4 treatments, with either standard drug or root and leaf extracts of *A. boonei*, and the parasitaemia suppressive effect of the extracts or standard drug at the end of day 4 treatments. The percentage parasitaemia was significantly (p<0.05) less in the groups administered 400, 600 and 800 mg/kg body wt/day concentrations of *A. boonei* extracts compared to the Control group, and the percentage suppression of parasitaemia in the same groups administered aqueous leaf, ethanolic leaf and root extracts of *A. boonei* was comparable to the standard drug after day 4 treatment.

**Table 1:** Percentage parasitaemia and chemosuppressive activities of aqueous and ethanolic leaf and root extracts of *A. boonie* in *P. berghei* infected mice (suppressive test)

Treatments	Parasitaemia (%)	Chemosuppressive activities
Baseline	0.00 ± 0.00^a1^	0.00 ± 0.00^a1^
Control	2.12 ± 6.41^b7^	0.00 ± 0.00^a1^
Artesunate	6.87 ± 1.53^a2^	67.67 ± 7.42^b5^
Aqueous leaf (400 mg/kg)	9.57 ± 2.03^a4^	54.66 ± 9.62^b4^
Aqueous leaf (600 mg/kg)	9.10 ± 2.00^a4^	57.00 ± 9.50^b4^
Aqueous leaf (800 mg/kg)	7.00 ± 1.75^a3^	66.33 ± 7.69^b5^
Aqueous Root (400 mg/kg)	31.17 ± 5.73^a2^	39.33 ±11.14^b3^
Aqueous Root (600 mg/kg)	22.57 ± 7.95^a8^	56.00 ± 15.31^b4^
Aqueous root (800 mg/kg)	15.00 ± 3.05^a6^	71.00 ± 5.51^b6^
Ethanolic leaf (400 mg/kg)	7.53 ± 2.41^a3^	86.33 ± 4.49^b7^
Ethanolic leaf (600 mg/kg)	6.67 ± 1.76^a2^	88.00 ± 3.22^b7^
Ethanolic leaf (800 mg/kg)	6.60 ± 2.44^a2^	88.33 ± 4.26^b7^
Ethanolic Root (400 mg/kg)	12.60 ± 3.81^a5^	77.33 ± 7.77^b6^
Ethanolic Root (600 mg/kg)	12.23 ± 1.19^a5^	78.00 ± 2.31^b6^
Ethanolic root (800 mg/kg)	7.87 ± 2.67^a3^	86.00 ± 4.58^b7^

All values expressed as mean ± standard error of mean. Different superscript letters on the same row indicates significance difference (*p<0.05*) in mean values among treatment indices. Different superscript numerals in the same column indicates significance difference (*p<0.05*) in mean values of extracts.

**Table 2:** Percentage parasitaemia and chemosuppresion of *P. berghei* in infected mice at day 4 post treatment with aqueous and ethanolic leaf and root extracts of *Alstonia boonei* (curative test)

Treatments (mg/Kg b. wt/day)	Parasitaemia (%)	Chemosuppressive activities
Baseline	0.00 ± 0.00^a1^	0.00 ± 0.00^a1^
Control	6.53 ± 8.43^b13^	0.00 ± 0.00^a1^
Artesunate	7.00 ± 0.58^a2^	88.33 ± 1.76^b4^
Aqueous leaf (400 mg/kg)	12.00 ± 1.73^a6^	81.67 ± 2.60^b4^
Aqueous leaf (600 mg/kg)	9.33 ± 1.86^a4^	85.66 ± 2.85^b4^
Aqueous leaf (800 mg/kg)	8.17 ± 1.01^a3^	87.66 ± 1.45^b4^
Aqueous Root (400 mg/kg)	24.00 ± 2.08^a12^	64.73 ± 2.99^b2^
Aqueous Root (600 mg/kg)	21.33 ± 4.18^a11^	66.40 ± 3.82^b2^
Aqueous root (800 mg/kg)	14.03 ± 2.31^a7^	73.00 ± 2.52^b3^
Ethanolic leaf (400 mg/kg)	12.93 ± 2.30^a6^	79.67 ± 3.53^b2^
Ethanolic leaf (600 mg/kg)	11.50 ± 3.33^a5^	87.33 ± 1.20^b4^
Ethanolic leaf (800 mg/kg)	7.67 ± 1.96^a2^	88.00 ± 3.06^b4^
Ethanolic Root (400 mg/kg)	19.60 ± 1.93^a10^	76.33 ± 2.33^b3^
Ethanolic Root (600 mg/kg)	18.43 ± 0.81^a9^	77.66 ± 0.88^b3^
Ethanolic root (800 mg/kg)	15.33 ± 1.20^a8^	81.67 ± 1.20^b4^

All values expressed as mean ± standard error of mean. Different superscript letters on the same row indicates significance difference (*p<0.05*) in mean values among treatment indices. Different superscript numerals in the same column indicates significance difference (*p<0.05*) in mean values of extracts.

### *Alstonia boonei* altered biochemical indices

The effects of the aqueous and ethanolic leaf and root extracts of *A. boonei* in *P. berghei* infected mice biochemical indices are summarized in Tables 3 – 7. The biochemical indices of alanine aminotransferase, aspartate aminotransferase, and creatinine of mice infected with *P. berghei* between 4 and 7 days treatment with extracts normalized the post-infection significantly (p<0.05) and insignificantly (p≥0.05) increases and alterations in the concentrations, which is comparable to the Artesunate (Tables 3 – 5). From the results, the parameters of alkaline phosphatase and total protein as observed after aqueous and ethanolic leaf and root extracts of *A. boonei* was administered demonstrated an insignificantly (p≥0.05) inconsistent alterations (Tables 6 and 7). The extract effects compared well to the Artesunate for the same duration of treatment.

**Table 3:** Effects of aqueous and ethanolic leaf and root extracts of *Alstonia boonei* treatments on alanine aminotransferase of *P. berghei* infected mice

Treatments(mg/Kg b. wt/day)	ALT (I/U)		
	BI	AI	AT
Baseline	23.00±0.87^a5^	23.00±0.87^a1^	22.17±0.88^a2^
Control	23.00±0.58^a5^	26.83±1.09^a4^	38.07±0.12^b6^
Artesunate	20.50±0.29^a2^	27.00±0.00^b5^	26.93±1.59^b5^
Aqueous leaf (400 mg/kg)	21.00±0.58^a3^	25.30±0.66^b3^	21.93±0.48^a1^
Aqueous leaf (600 mg/kg)	21.67±0.88^a3^	27.00±0.87^b5^	22.40±0.45^a2^
Aqueous leaf (800 mg/kg)	21.00±0.00^a3^	24.00±0.70^c2^	23.70±0.46^b3^
Aqueous Root (400 mg/kg)	20.83±0.60^a2^	26.03±0.55^c2^	21.73±1.17^b1^
Aqueous Root (600 mg/kg)	23.63±1.68^b5^	26.83±1.42^c4^	21.90±1.40^a1^
Aqueous root (800 mg/kg)	22.00±1.16^a4^	25.33±0.83^c3^	23.90±1.43^b3^
Ethanolic leaf (400 mg/kg)	19.67±0.67^a1^	27.86±0.94^c5^	24.50±0.40^b4^
Ethanolic leaf (600 mg/kg)	22.20±1.11^a4^	24.56±0.56^c2^	23.53±1.35^b3^
Ethanolic leaf (800 mg/kg)	19.00±0.58^a1^	26.07±0.48^c4^	23.80±0.25^b3^
Ethanolic Root (400 mg/kg)	22.00±0.00^a4^	25.17±0.73^c3^	24.23±0.29^b4^
Ethanolic Root (600 mg/kg)	22.33±1.20^a4^	25.90±0.08^c3^	24.57±1.11^b4^
Ethanolic root (800 mg/kg)	21.67±0.88^a3^	25.67±1.20^c3^	22.30±0.35^b2^

All values expressed as mean ± standard error of mean. Different superscript letters on the same row indicates significance difference (*p<0.05*) in mean values among different treatment periods. Different superscript numerals in the same column indicates significance difference (*p<0.05*) in mean values of extract. BI: Before Inoculation; AI: After Inoculation; AT: After Treatment.

**Table 4:** Effects of aqueous and ethanolic leaf and root extracts of *Alstonia boonei* treatments on aspartate aminotransferase of *P. berghei* infected mice

Treatments(mg/Kg b. wt/day)	AST (I/U)		
	BI	AI	AT
Baseline	26.97 ± 0.55^a3^	26.97 ± 0.55^a1^	26.97 ± 0.55^a2^
Control	27.00 ± 0.58^a4^	35.67 ± 1.45^b3^	50.07 ± 0.58^c3^
Artesunate	25.23 ± 0.75^a2^	42.67 ± 0.33^c6^	27.37 ± 1.31^b3^
Aqueous leaf (400 mg/kg)	26.83 ± 1.92^a3^	44.00 ± 3.27^c8^	30.77 ± 0.15^b5^
Aqueous leaf (600 mg/kg)	27.30 ± 0.30^a4^	43.33 ± 3.71^c7^	29.33 ± 1.86^b4^
Aqueous leaf (800 mg/kg)	27.53 ± 0.82^a4^	38.67 ± 3.84^b4^	27.37 ± 1.31^a3^
Aqueous Root (400 mg/kg)	26.33 ± 1.20^a3^	41.33 ± 1.86^c5^	39.03 ± 0.58^b7^
Aqueous Root (600 mg/kg)	24.83 ± 0.73^a1^	47.00 ± 1.53^c10^	31.23 ± 1.74^b6^
Aqueous root (800 mg/kg)	25.70 ± 0.30^a2^	45.33 ± 0.33^b9^	25.93 ± 1.18^a1^
Ethanolic leaf (400 mg/kg)	26.00 ± 0.58^a3^	42.37 ± 1.44^c2^	29.00 ± 1.00^b4^
Ethanolic leaf (600 mg/kg)	26.00 ± 1.53^a3^	50.00 ± 0.00^c12^	29.00 ± 0.58^b4^
Ethanolic leaf (800 mg/kg)	26.07 ± 0.07^a3^	48.00 ± 2.00^b11^	26.33 ± 0.62^a2^
Ethanolic Root (400 mg/kg)	25.33 ± 0.33^a2^	34.67 ± 0.67^c2^	30.03 ± 0.55^b5^
Ethanolic Root (600 mg/kg)	28.00 ± 0.06^a5^	35.67 ± 0.33^c3^	29.37 ± 0.68^b4^
Ethanolic root (800 mg/kg)	26.10 ± 0.10^a3^	34.67 ± 1.20^c2^	29.16 ± 0.44^b4^

All values expressed as mean ± standard error of mean. Different superscript letters on the same row indicates significance difference (*p<0.05*) in mean values among different treatment periods. Different superscript numerals in the same column indicates significance difference (*p<0.05*) in mean values of extract. BI: Before Inoculation; AI: After Inoculation; AT: After Treatment.

**Table 5:** Effects of aqueous and ethanolic leaf and root extracts of *Alstonia boonei* treatments on creatinine level of *P. berghei* infected mice

Treatments(mg/Kg b. wt/day)	Creatinine (mg/dl)		
	BI	AI	AT
Baseline	0.73 ± 0.88^b2^	0.63 ± 0.09^a1^	0.73 ± 0.09^b1^
Control	0.67 ± 0.88^a1^	2.03 ± 0.34^b3^	3.03 ± 0.03^c3^
Artesunate	0.77 ± 0.12^a2^	2.27 ± 0.29^b3^	0.73 ± 0.03^a1^
Aqueous leaf (400 mg/kg)	0.80 ± 0.06^a3^	1.83 ± 0.12^b2^	1.30 ± 0.17^b2^
Aqueous leaf (600 mg/kg)	0.66 ± 0.07^a1^	2.17 ± 0.32^c3^	1.23 ± 0.15^b12^
Aqueous leaf (800 mg/kg)	0.83 ± 0.09^a3^	2.33 ± 0.29^b3^	0.80 ± 0.06^a1^
Aqueous Root (400 mg/kg)	0.73 ± 0.03^a2^	1.43 ± 0.34^b2^	1.06 ± 0.23^b2^
Aqueous Root (600 mg/kg)	0.63 ± 0.09^a1^	2.00 ± 0.12^b3^	0.77 ± 0.12^a1^
Aqueous root (800 mg/kg)	0.83 ± 0.09^a3^	1.87 ± 0.18^b2^	0.70 ± 0.15^a1^
Ethanolic leaf (400 mg/kg)	0.77 ± 0.03^a2^	2.00 ± 0.15^b3^	1.73 ± 0.12^a2^
Ethanolic leaf (600 mg/kg)	0.60 ± 0.06^a1^	2.23 ± 0.12^c3^	1.17 ± 0.09^b2^
Ethanolic leaf (800 mg/kg)	0.60 ± 0.00^a1^	2.20 ± 0.15^c3^	1.00 ± 0.58^b2^
Ethanolic Root (400 mg/kg)	0.67 ± 0.03^a1^	2.13 ± 0.17^c3^	1.00 ± 0.12^b2^
Ethanolic Root (600 mg/kg)	0.77 ± 0.09^a2^	2.30 ± 0.00^b3^	0.87 ± 0.09^a1^
Ethanolic root (800 mg/kg)	0.80 ± 0.06^a3^	2.20 ± 0.06^b3^	0.80 ± 0.06^a1^

All values expressed as mean ± standard error of mean. Different superscript letters on the same row indicates significance difference (*p<0.05*) in mean values among different treatment periods.

Different superscript numerals in the same column indicates significance difference (*p<0.05*) in mean values of extract. BI: Before Inoculation; AI: After Inoculation; AT: After Treatment.

**Table 6:** Effects of aqueous and ethanolic leaf and root extracts of *Alstonia boonei* treatments on alkaline phosphatase of *P. berghei* infected mice

Treatments(mg/Kg b. wt/day)	ALP (I/U)		
	BI	AI	AT
Baseline	41.93 ± 0.98^a11^	42.97 ± 0.55^a3^	42.97 ± 0.55^a4^
Control	38.33 ± 2.40^a9^	44.33 ± 1.20^b5^	55.23 ± 1.61^c6^
Artesunate	40.33 ± 2.60^a10^	43.97 ± 0.55^c4^	42.97 ± 0.55^b4^
Aqueous leaf (400 mg/kg)	38.03 ± 1.16^a9^	44.33 ± 0.03^c1^	43.13 ± 0.65^b5^
Aqueous leaf (600 mg/kg)	36.30 ± 1.72^a7^	41.33 ± 0.88^b2^	43.30 ± 0.70^c5^
Aqueous leaf (800 mg/kg)	37.67 ± 2.33^a8^	43.67 ± 1.20^c4^	42.24 ± 1.28^b4^
Aqueous Root (400 mg/kg)	37.53 ± 351^a8^	40.66 ± 0.33^b1^	40.56 ± 4.96^b1^
Aqueous Root (600 mg/kg)	29.67 ± 3.84^a1^	40.33 ± 0.88^b1^	41.50 ± 1.18^c3^
Aqueous root (800 mg/kg)	34.00 ± 1.15^a5^	40.67 ± 0.67^b1^	42.43 ± 1.26^c4^
Ethanolic leaf (400 mg/kg)	31.77 ± 1.20^a2^	43.33 ± 0.88^c4^	37.00 ± 0.06^b1^
Ethanolic leaf (600 mg/kg)	32.67 ± 1.77^a3^	41.66 ± 1.20^c2^	37.03 ± 0.61^b1^
Ethanolic leaf (800 mg/kg)	31.07 ± 0.58^a2^	42.27 ± 0.69^c3^	39.47 ± 0.33^b2^
Ethanolic Root (400 mg/kg)	33.00 ± 1.53^a4^	42.33 ± 0.26^c3^	40.33 ± 1.33^b2^
Ethanolic Root (600 mg/kg)	35.00 ± 1.53^a6^	41.00 ± 0.58^c2^	40.90 ± 0.95^b2^
Ethanolic root (800 mg/kg)	32.00 ± 1.15^a3^	42.00 ± 0.58^c3^	41.93 ± 0.61^b3^

All values expressed as mean ± standard error of mean. Different superscript letters on the same row indicates significance difference (*p<0.05*) in mean values among different treatment periods.

Different superscript numerals in the same column indicates significance difference (*p<0.05*) in mean values of extract. BI: Before Inoculation; AI: After Inoculation; AT: After Treatment.

**Table 7:** Effects of aqueous and ethanolic leaf and root extracts of *Alstonia boonei* treatments on total protein of *P. berghei* infected mice

Treatments(mg/Kg b. wt/day)	Total Protein (g/dl)		
	BI	AI	AT
Baseline	4.80 ± 0.06^b5^	4.53 ± 0.12^a2^	4.80 ± 0.06^b1^
Control	4.83 ± 0.15^b5^	4.43 ± 0.07^a2^	7.17 ± 0.09^c4^
Artesunate	4.70 ± 0.06^b4^	4.50 ± 0.06^a2^	4.73 ± 0.09^b1^
Aqueous leaf (400 mg/kg)	4.60 ± 0.06^b3^	4.43 ± 0.07^a2^	4.90 ± 0.06^c1^
Aqueous leaf (600 mg/kg)	4.70 ± 0.05^b4^	4.07 ± 0.33^a2^	4.87 ± 0.09^c2^
Aqueous leaf (800 mg/kg)	4.70 ± 0.06^b4^	4.20 ± 0.00^a2^	4.80 ± 0.06^c1^
Aqueous Root (400 mg/kg)	4.43 ± 0.09^b1^	3.70 ± 0.00^a1^	5.30 ± 0.06^c2^
Aqueous Root (600 mg/kg)	4.53 ± 0.12^b2^	3.50 ± 0.15^a1^	5.27 ± 0.09^c2^
Aqueous root (800 mg/kg)	4.53 ± 0.20^b2^	3.67 ± 0.12^a1^	4.53 ± 0.12^b1^
Ethanolic leaf (400 mg/kg)	4.46 ± 0.03^b1^	3.77 ± 0.09^a1^	5.63 ± 0.33^c2^
Ethanolic leaf (600 mg/kg)	4.45 ± 0.06^b1^	3.70 ± 0.15^a1^	5.60 ± 051^c3^
Ethanolic leaf (800 mg/kg)	4.46 ± 0.00^b1^	3.70 ± 0.21^a1^	4.47 ± 0.12^b1^
Ethanolic Root (400 mg/kg)	4.70 ± 0.06^b4^	4.00 ± 0.00^a2^	5.10 ± 0.06^c2^
Ethanolic Root (600 mg/kg)	4.97 ± 0.07^b6^	4.23 ± 0.09^a2^	4.90 ± 0.06^b1^
Ethanolic root (800 mg/kg)	4.77 ± 0.38^c4^	4.20 ± 0.10^a2^	4.67 ± 0.09^b1^

All values expressed as mean ± standard error of mean. Different superscript letters on the same row indicates significance difference (*p<0.05*) in mean values among different treatment periods. Different superscript numerals in the same column indicates significance difference (*p<0.05*) in mean values of extract. BI: Before Inoculation; AI: After Inoculation; AT: After Treatment.

## Discussion

### Mean survival time of *P. berghei* infected mice

Mean survival is a statistic that refers to how long patients survive with a disease in general or after a certain treatment. In this study, *P. berghei* infected mice treated with aqueous and ethanolic leaf and root extracts of *A. boonei* had similar MST that was statistically different and higher than infected and untreated mice. This indicated that the aqueous and ethanolic leaf and root extracts of *A. boonei*, as well as the standard drug, were effective in suppressing the level of parasitemia in the infected mice. In a comparative study of genotoxicity and anti-plasmodial activities of stem and leaf extracts of *A. boonei* in malaria-infected mice, chloroquine had the highest MST followed by *A. boonei* stem extract^30^.

### *Alstonia boonei* suppressed parasitaemia

*Plasmodium berghei* has been used in predicting treatment outcomes of any suspected anti-malaria agent because of its high sensitivity to malaria drug-like chloroquine, artesunate etc., thus making it the appropriate parasite for this study^31^, and has been used in studying the anti-malaria potentials in mice^32^. The suppressive test is a standardized test commonly used for anti-malaria screening. It is used for the determination of percentage inhibition of parasitaemia^5^. The result of this study indicated that the aqueous and ethanolic leaf and root extracts of *A. boonei* exhibited some activities against *P. berghei*, especially in ethanolic leaf extracts. The chemo-suppressive effect of the extracts occurred in a dose-dependent manner in the different extracts. The highest suppressive effect was observed with the standard drug (artesunate). However, the value was similar to that observed for extract dose of 800 mg/kg/day. In this study, the result of the curative effect of the plant extracts equally showed a concentration-dependent activity. The highest curative effect of the plant extracts was observed when the highest dose (800 mg/kg/body wt/day) was administered. This was consistent with the findings of Onwusonye and Uwakwe^19^ who also recorded the highest curative effect for the root bark of the plant extract when the same dose was administered. There were significant decreases in parasite density in the treated groups compared to the untreated group. The present finding agreed with the report of Matsuoka et al.^33^ that when a standard anti-malaril drug was used in mice infected with *P. berghei*, it suppressed the parasitaemia to a non-detectable level. The antimalarial activity of *A. boonei* aqueous and ethanolic leaf and root extracts could be attributed to the presence of some phytochemicals in the plant^16^. Both aqueous and ethanolic extracts had almost similar secondary metabolites except for the presence of saponins only in the ethanolic extract. General glycosides, flavonoids, terpenoids and steroids, and alkaloids were present in both aqueous and ethanolic leaf and root extracts of *A. boonei*, while carotenoids, coumarins, anthraquinones, anthraquinones glycosides and cyanogenetic glycosides were absent in both extracts^34^. Specifically, the parasitaemia suppression effect of the extract may be attributed to the presence of alkaloids^35^,^36^.

### *Alstonia boonei* altered biochemical indices

In this study, administration of A. boonei extracts and artesunate normalized the activities of aspartate aminotransferase and alanine aminotransferase enzymes found in various parts of the body. Elevated amounts of these enzymes in the blood may signal a health problem^37^. AST and ALT test is commonly used to check for liver disease, to monitor liver disorder, to ascertain treatment efficacy and to make sure that medications are not causing liver damage. After inoculation of *P. berghei*, there was a significant increase in AST of infected mice (Control group, Artesunate group and the groups treated with different concentration of extracts) when compared to the Baseline group. This is as a result of degeneration changes in the hepatocytes due to the infection by *P. berghei* that have altered the activity of enzymes. After treatment, it was observed that levels of AST and ALT were significantly higher (p<0.05) in the Control group when compared to the Artesunate group and the groups treated with extracts. This may be as a result of inducing the activity of the enzyme by the extracts and the standard drug and was consistent with the report of Momoh et al.^5^ that the AST and ALT values of Control group were higher due to the normality of the enzyme activity by the extracts. From the present study, there was a significant increase (p<0.05) in plasma creatinine as a result of damage caused by the continuous multiplication of the parasite when compared to Baseline groups, Artesunate group and the groups treated with different concentration of extracts. After inoculation, there was significant (p<0.05) increase in alanine phosphatase in the Control group, Artesunate group and the groups treated with different concentration of the extracts when compared to the Baseline in all the extracts except in aqueous leaves extract as a result of infection by the *P. berghei*. After treatment, ALP of the Control group had significantly (p<0.05) higher ALP when compared to the Baseline group, Artesunate group and the groups trated with different concentration of the extracts. This increase in ALP is associated with liver damage which was slightly normalized in the group treated with the extracts and the standard drug. This finding was in agreement with the reports of Momoh et al.^5^, Halim et al.^38^ and Momoh and Manuwa^39^ who reported that an increase in ALP level was as a result of liver damage. Total protein also is a biochemical test for measuring the total amount of protein in the serum. In this study, it was observed that after treatment, there was a decrease in the total protein level of the Control group when compared to other groups. This may be due to the reduction in protein synthesis. Since malaria destroys cells that are responsible for protein synthesis, these findings agreed with an earlier report that chronic infections are autoimmune diseases that reduced protein synthesis^40^.

Furthermore, the findings of this study were in agreement with the finding of studies on therapeutic potentials, chemopreventive and remediation effect of Adansonia digitata stem bark extracts in rodent malaria^41^,^42^. They reported that *A. digitata* stem back aqueous and methanolic extracts showed a significant dose-dependent increase percentage chemosupression/clearance, PCV and a significant decrease in percentage parasitemia at the two doses administered after established infection^41^. Methanolic extract (400 mg/kg) exhibited the highest chemosupressive activity. The extract significantly reduced the degree of tissue peroxidation, increased the level of reduced glutathione (GSH), superoxide dismutase and catalase activity. Furthermore, the extracts reduced serum tumour necrosis factor-alpha (TNF-α) and C-reactive protein (CRP) concentrations and serum and tissue ALP activity^42^.

The normalization of liver enzyme activity in *A. boonei* extract-treated mice infected with *P. berghei* has been attributed to rich vitamin, mineral and phytochemical contents of *A. boonei*^16^. Considering the phytochemicals, flavonoids are potent water-soluble antioxidants and free radical scavengers that prevent oxidative cell damage, have high anticancer activity and also lower the risk of heart diseases^43^. Saponin neutralizes the effect of some harmful gut enzymes, build the immune system and promoting wound healing. Alkaloids have been linked to analgesic, antispasmodic and bactericidal activities, and tannins are reported to hasten the healing of wounds and inflamed mucous membrane^44^. Cardiac steroids have been linked to the treatment of congestive heart failure by increasing the force of heart contraction (positive inotropic activity) in patients with heart failure^44^. The presence of these phytochemicals in *A. boonei* supports its medicinal use.

## Conclusion

The extracts potentials of *A. boonei* leaf and root were dependent on both dosage and duration, and have demonstrated satisfactory normalization efficacy to biochemical indices in malaria treatment.
